# Formulation of inhalable lipid-based salbutamol sulfate microparticles by spray drying technique

**DOI:** 10.1186/2008-2231-22-50

**Published:** 2014-06-11

**Authors:** Zahra Daman, Kambiz Gilani, Abdolhossein Rouholamini Najafabadi, Hamid Reza Eftekhari, Mohammad Ali Barghi

**Affiliations:** 1 Aerosol Research Laboratory, Department of Pharmaceutics, School of Pharmacy, Tehran University of Medical Sciences, Tehran, Iran; 2 Medicinal Plants Research Center, Tehran University of Medical Sciences, Tehran, Iran; 3 XRD Research Laboratory, School of Sciences, Tehran University, Tehran, Iran

**Keywords:** Dry powder inhalation, Spray drying, Salbutamol sulfate, Solid lipid microparticles, Particle engineering, L-leucine

## Abstract

**Background:**

The aim of this work was to develop dry powder inhaler (DPI) formulations of salbutamol sulfate (SS) by the aid of solid lipid microparticles (SLmPs), composed of biocompatible phospholipids or cholesterol.

**Methods:**

The SLmPs were prepared by using two different solvent systems (ethanol and water-ethanol) and lipid carriers (dipalmitoylphosphatidylcholine (DPPC) and cholesterol) with/without L-leucine in the spray drying process. The spray-dried microparticles were physically-mixed with coarse lactose monohydrate in order to make our final DPI formulations and were investigated in terms of physical characteristics as well as in vitro drug release profile and aerosolization behavior.

**Results:**

We observed significant differences in the sizes, morphologies, and in vitro pulmonary depositions between the formulations. In particular, the SS-containing SLmPs prepared with water-ethanol (30:70 v/v) solution of DPPC and L-leucine which had then been blended with coarse lactose (1:9 w/w) exhibited the highest emitted dose (87.9%) and fine particle fraction (42.7%) among the formulations. In vitro drug release study indicated that despite of having a significant initial burst release for both cholesterol and DPPC-based microparticles, the remained drug released more slowly than the pure drug.

**Conclusion:**

This study demonstrated the potential of using lipid carriers as well as L-leucine in DPI formulations of SS to improve its aerosolization behavior and retard the release profile of the drug.

## Background

Local administration of drugs to the lungs presents several advantages, especially, in patients with specific pulmonary diseases, such as asthma, pulmonary infections or lung cancer. The advantages include reduction in systemic side effects, elevated drug concentration at the site of action, and reduction in amount of drug administered to the patient compared to traditional routs [1-3]. In this regard, the area of particle engineering is becoming increasingly attractive for the development of more efficient inhaled therapeutics.

One of the attractive applications of particle engineering is to develop a sustained release (SR) formulation by using suitable carriers, a kind of formulation that has not been marketed yet, despite active research conducted on this subject. A SR formulation will provide the active drug over an extended duration of time, and therefore may improve therapy by enhancing the compliance of the patients. In such formulations, it is expected that the overall amount of drug and the side effects will be reduced [4-6]. However, the efforts for finding suitable, non-toxic excipients, which can produce a desired drug release profile and improve the respirable fraction of the inhaled particles to maximize drug deposition into smaller airways are continuous and extensive.

One approach to SR delivery to the respiratory tract utilizes liposomal formulations. Liposomes are promising vehicles for pulmonary drug delivery owing to their capacity to increase drug retention time and reduce the toxicity of drugs after administration [7,8]. Several factors such as the composition of lipids and the size of liposomes can affect the performance of the system [9-11]. Many studies have shown the applicability of liposomes in lung delivery of a large variety of drugs such as cytotoxic agents, anti-asthma drugs, antimicrobial agents, and drugs for systemic action like insulin and other proteins [4,10]. However, there are some disadvantages about liposomal vehicles that limits their application as commercial formulations such as high production cost and instability during storage even at low temperatures [12], and nebulization [13,14] that can cause premature release of the entrapped drug. The latter problem has been reported even about the dry powder formulations prepared by jet milling micronization of lyophilized liposomes, which deleteriously affected their integrity [15].

Another approach for development of an inhalable SR formulation is to produce solid lipid microparticles (SLmPs). It has been suggested that SLmPs offer high tolerability in the pulmonary tract, as they are mainly made of biocompatible and biodegradable materials [16,17]. Moreover, they possess several other advantages compared to traditional vehicles such as polymeric drug carriers, micelles or liposomes, including more physiochemical stability, incorporation of both lipophilic and hydrophilic drugs, low large-scale production cost and having no significant biotoxicity [16-19]. Phospholipids and cholesterol have been previously used in inhalation formulations as solid lipid carriers or fillers to improve drug targeting to the lung. The prepared SLmPs presented spherical shapes, reduced agglomeration tendency and high fine particle fraction (FPF) [17,20].

Spray drying is an attractive solidification technique in the field of respiratory drug delivery, with respect to its relative simplicity, availability of large-scale equipment, capability to produce homogenous particle size distribution, and ability to control various parameters that optimize the particulate product characteristics such as size, size distribution, shape, morphology and density [21-23]. Therefore, it can be used as a suitable technology to produce dry powder inhaler (DPI) products, which possess several advantages over pressurized metered dose inhalers (pMDI), such as being breath-activated and having no requirement of any propellant [24].

Thus, the aim of this study was to design SLmPs using cholesterol or dipalmitoylphosphatidylcholine (DPPC) by spray drying method. The idea was emerged from the potential ability of these excipients to entrap both water-soluble and water-insoluble drugs, as well as providing a prolonged local drug release [6,16]. Moreover, the safety issue of these SLmPs over other vehicles was a key consideration in our design process, since they are mainly made from endogenous materials [25,26]. For this purpose, we chose to work with SS, a short acting beta2-adrenoceptor stimulant with plasma half-life of 4–6 hours, which requires frequent dosing for daily management of asthma. A SR preparation of this agent is desirable approach to improve therapy of asthma, especially in non-compliant patients and also for covering the nocturnal decline of the drug [27], when administered at the bed time.

Aside from SR properties, an effective DPI formulation should offer optimum particle characteristics to achieve high FPF and reduce the central deposition in pulmonary airways. In other words, a suitable DPI formulation should have the ability to reach deep lung regions and disperse adequately within the airflow of the patient. Indeed, decreasing of both particle size and density can be achieved by spray drying technique in order to generate particles with satisfactory respirable fraction [23]. However, the dispersibility of the particles is another factor that has to be taken into consideration. The particle aggregation associated with cohesive forces between them can be regulated using excipients such as coarse crystalline lactose, which is currently serving as the drug carrier and also the bulking agent in most available DPI products [23]. Usually, drug particles and such excipients are combined in a physical blending process during which the microparticles are attached to the surface of the carrier. Therefore, our final DPI formulations consisted of physically-mixed SLmPs with large coarse lactose carrier particles. To aid dispersibility, it has been also proven that co-spray drying of simple amino acids, especially the hydrophobic ones such as L-leucine, can improve dispersion of the powder and may enhance the fraction of respirable particles [28]. Thus, we used this amino acid in our spray drying process to evaluate its effects on the aerodynamic performance of the resultant DPI formulation.

In the present study, the obtained SLmPs were further characterized for their physical properties, in vitro aerosolization behavior, and their potential of being a SR delivery system.

## Methods

### Materials

SS was supplied as micronized powder from Darupakhsh (Iran). Cholesterol was purchased from Merck (Germany), and the phospholipid, DPPC, was supplied from Lipoid (Germany). Inhalation grade lactose (Pharmatose 325 M) with D50% of about 60 μm was obtained from DMV Internationals (The Netherlands). Other chemical reagents and solvents including the HPLC grade ones were purchased from either Merck or Sigma. L-Leucine was also supplied from Merck (Germany).

### Preparation of the lipid-based microparticles

The SLmPs were prepared, at laboratory scale, by spray drying method using a Büchi Minispray dryer B-191-a from Büchi Laboratory-Technique (Switzerland). In this study, we decided to improve the drying efficiency of the lipid excipients by using a jacketed cyclone with cold-water circulation, to cool down the cyclone separator wall and thus decrease the lipid particles’ adhesion and agglomeration.

Two different types of formulations were spray dried for the preparation of SLmPs. The first type was prepared by dispersing the SS microparticles within an ethanol solution of the hydrophobic excipients, cholesterol or DPPC. The suspensions were sonicated for 10 min before spray drying to ensure the adequate dispersion of the drug. The second type of formulations was obtained from spray drying of water-ethanol (30:70 v/v) solution of the drug and the lipid materials. Details are shown in Table 1.

**Table 1:** Composition of different spray-dried formulations

**Formulation number**	Drug conc. (%)*	**Excipients**	**Solvent system**	**Inlet temperature (°C)**
1	12.5	cholesterol	Ethanol	80
2	25	cholesterol	Ethanol	80
3	37.5	cholesterol	Ethanol	80
4	37.5	DPPC	Ethanol	80
5	37.5	cholesterol	Water/Ethanol	100
6	37.5	DPPC	Water/Ethanol	100
7	37.5	DPPC + Leucine	Water/Ethanol	100

*Percentage of the total solid content (w/w).

The spray drying conditions were as following: Solid content, 5% w/v; Nozzle size, 0.5 mm; Inlet temperature, 80/100°C (depending on the solvent system); Outlet temperature, 54/65°C (depending on the inlet temperature); Spraying air flow rate, 800 L/h; Feed rate, 0.2 g/min; Cold water circulation in the jacketed cyclone, 0°C.

Furthermore, as shown in Table 1, L-leucine was co-spray dried at the amount of 10% w/w with respect to the solid content with water-ethanol solution of DPPC and SS. Finally, all the obtained formulations were physically blended with inhalation grade lactose monohydrate (Pharmatose^®^ 325 M) at a ratio of 1:9 w/w in a Turbula mixer from Dorsa Novin (Iran) for 60 min at a low speed (46 rpm).

### Determination of SS content

Quantification of CIP was conducted by HPLC using a mobile phase consisting of water, methanol and phosphate buffer (pH 2.8) in the ratio of 60:20:20 at a flow rate of 1 mL/min. The phosphate buffer was prepared by dissolving 2.625 g ammonium phosphate in 50 mL purified deionized water, adding 2.8 mL of phosphoric acid (85%) and diluting to 100 mL with purified deionized water. The HPLC system consisted of a pump (Waters 600E, Millipore, USA), a C18 Tracer Excel column (15 × 0.46 cm, 5 μm, Spain) and a UV detector (Waters 486, USA) at 276 nm. Bamethan sulfate (to final concentration of 10 μg/mL) was added as the internal standard to each sample just before analysis. From the relative area under the peak, linearity (R2 = 0.999) was achieved using standard aqueous solutions of SS between 0.5 and 50 μg/mL.

For all the prepared DPI formulations, the content uniformity was evaluated by taking 10 random samples, each weighing 10 mg powder which were subjected to lipid extraction by adding 1.5 mL chloroform to each one and centrifugation at 37565 × g for 20 min. The recovered drug was diluted with mobile phase before being subjected to HPLC analysis. Mixtures with relative standard deviation values of less than 10%, as recommended by The United States Pharmacopeia, were considered to be satisfactorily mixed.

### Particle size measurement

The size distribution of the microparticles was determined by laser diffraction method using Malvern Mastersizer X (UK) after the formulations had been dispersed in appropriate medium (saturated solution of SS in water) and sonicated for 2 min. The geometrical diameter was expressed as volume median diameter (D50%). Also the Span values of formulations were defined as $\frac{D90\%-D10\%}{D50\%}$, which represents the breadth of the particle distribution. Each measurement was repeated in triplicate.

### Scanning electron microscopy

Particle morphology was observed by scanning electron microscopy (SEM) using Philips XL30 equipment (The Netherlands). The samples were coated with gold under relative vacuum by means of Bal-Tec/SCDOOS sputter coater (Switzerland) and were examined under an accelerating voltage of 25 kv.

### Determination of true density

The density was assessed with Quantachrome helium pycnometer (USA). The basis of this method is on putting the sample of known mass into a cell of known volume. Briefly, when helium penetrates into the cell at a vacuum, it occupies the entire volume of the cell, so the actual volume of the sample can be determined since the volume of the cell is known. So, the actual densities of the samples were accurately calculated. Each sample was analyzed thrice.

### Aerosol performance of SLmPs

The in vitro pulmonary deposition of the powders was determined by Twin Stage Impinger (TSI) glass apparatus from Copley Scientific (UK). A dry powder formulation device, Novartis Cyclohaler^®^ (Switzerland), was filled with a hard gelatin capsule loaded with 10 mg of each formulation. On the other hand, the mobile phase was introduced to stage 1 (7 mL) and stage 2 (30 mL) of the TSI. Once the assembly had been checked to be tight and vertical, the Cyclohaler^®^ had been inserted to the rubber mouthpiece attached to the throat part of the impinger. The test was operated at 60 L/min for 4 s. The flow rate was achieved using a rotary vein pump from Copley Scientific (UK). After the operation, the impinger components had been washed into separate volumetrics (25 mL for the throat and stage 1, and 50 mL for the device and stage 2) with the same solution. Their contents were assayed for SS, after the lipid had been extracted with certain ratio of chloroform.

Fine particle dose (FPD) was considered as the amount of drug deposited in stage 2 (dae < 6.4 μm). The emitted dose (ED) was determined as a percent of total powder exiting the capsule and the device. The FPF was calculated as the percent of the ratio of FPD to the total amount of drug emitted per capsule.

### Determination of drug release from SLmPs

Since the volume of surface liquid in the respiratory tract is relatively low, the conventional European Pharmacopeia methods cannot be used for exact evaluation of dissolution behavior of inhaled drugs due to their large volumes of dissolution media (900–1000 mL) [29]. Hence we used a dispersion method to measure in vitro release of the drug from SLmPs. Briefly, 10 mg of each formulation was suspended individually in 10 mL phosphate buffered saline (PBS, PH = 7.4) in test tubes and incubated in a shaker (Grant instruments, Cambridge, England) at 37°C on 50 rpm. At certain time intervals of 0.25, 0.5, 1, 2, 4, 8, and 12 hours, three tubes were picked and individually assayed for SS after being filtered. The mean value of the tree tubes for each time interval was calculated, and plotted as cumulative amount of SS released over 12 hours.

### Statistical analysis

Data for all measurements were considered as the mean ± standard deviation (SD) of at least three separate experiments. One-way analysis of variance (ANOVA) test was used for statistical comparison of the results while p < 0.05 was considered significant in all cases.

## Results and discussion

Different powder compositions were formulated using the spray drying technique, with the aim of studying the influence of lipid composition and the solvent type on the physiochemical properties and the aerosolization behavior of the powders. Table 1 gives an overview of all the prepared powder formulations. It should be mentioned that the content uniformity test was conducted for both spray-dried formulations and the physical blends, using a conventional invasive sampling method. The active drug content was quantified by HPLC, and ranged between 95 ± 2% and 103 ± 3% for different formulations.

### Evaluation of physiochemical properties of aerosol particles

The particle size characteristics of the formulations are summarized in Table 2. The results showed that for the same lipid and solvent composition of the formulations (cholesterol in ethanol), the percentage of SS in the suspensions used for spray drying had no significant effect on the size of resultant SLmPs (p > 0.05). Additionally, the D50% of the spray dried formulations obtained from ethanol suspension of the drug were shown to be dependent on the type of lipid component, which was much smaller for DPPC-based microparticles than cholesterol (p < 0.05). Changing the solvent from ethanol to water-ethanol (30:70 v/v) resulted in an increase in D50% values of both DPPC and cholesterol-based particles (p < 0.05). It seems that the enhancement in the inlet temperature of spray drying process has contributed to the particle size enlargement, as it was previously proven that adding in temperature will lead to increase in the diameter of particles [30,31]. Furthermore, the laser diffraction particle size analysis showed that co-spray drying of L-leucine with DPPC and SS did not significantly change the particle size distribution with respect to the counterpart sample without L-leucine (p > 0.05).

**Table 2:** Particle size measurement obtained by laser diffraction method (mean ± SD)

**Formulation number**	Drug conc. (%)*	**Excipients**	**Solvent system**	**Inlet temp. (°C)**	**D50%**	**Span**
1	12.5	cholesterol	Ethanol	80	3.23 ± 0.48	3.19
2	25	cholesterol	Ethanol	80	5.04 ± 0.66	1.75
3	37.5	cholesterol	Ethanol	80	4.16 ± 0.32	1.66
4	37.5	DPPC	Ethanol	80	1.42 ± 0.15	0.87
5	37.5	cholesterol	Water-Ethanol	100	7.32 ± 0.28	2.26
6	37.5	DPPC	Water-Ethanol	100	4.02 ± 0.18	2.54
7	37.5	DPPC + Leucine	Water-Ethanol	100	4.04 ± 0.25	2.23
C_1_	100	-	Ethanol	80	3.70 ± 0.13	2.47
C_2_	100	-	Water-Ethanol	100	5.83 ± 0.21	1.82

*Percentage of the total solid content (w/w).

**Figure 1:**
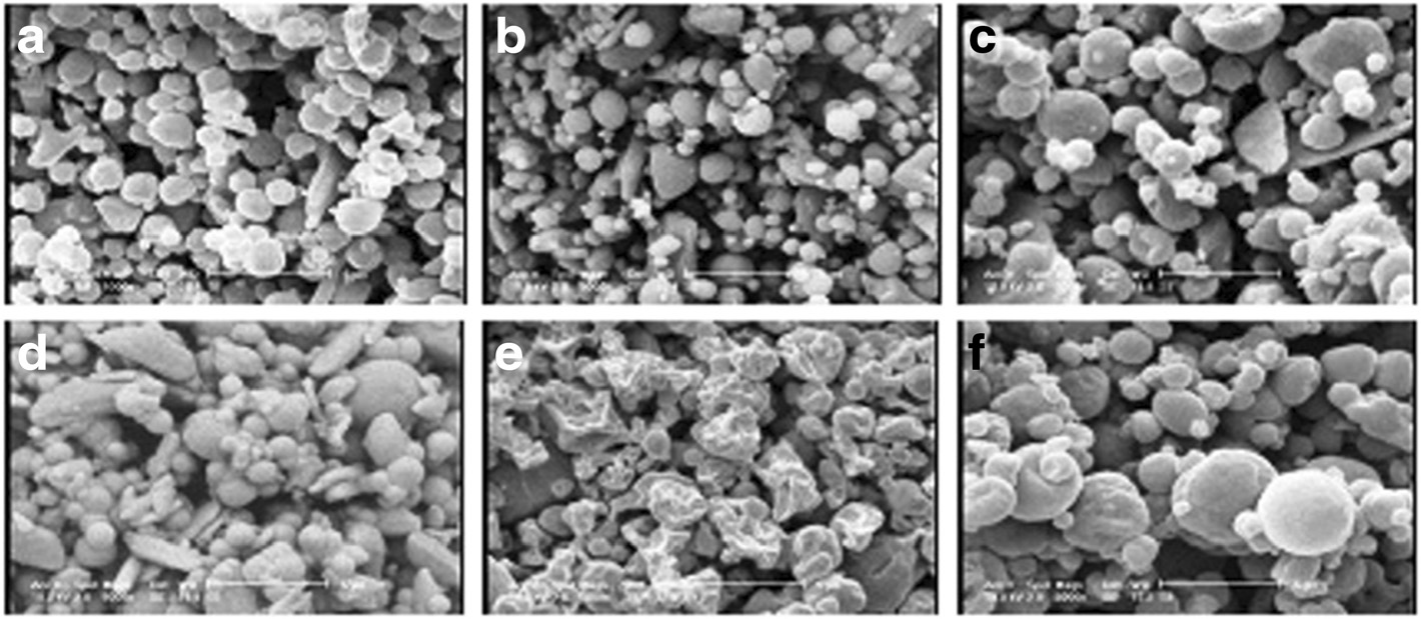
Scanning electron micrographs of SLmPs containing salbutamol sulfate in different formulations: a) F2, b) F3, c) F5, d) F4, e) F6, f) F7.

**Figure 2:**
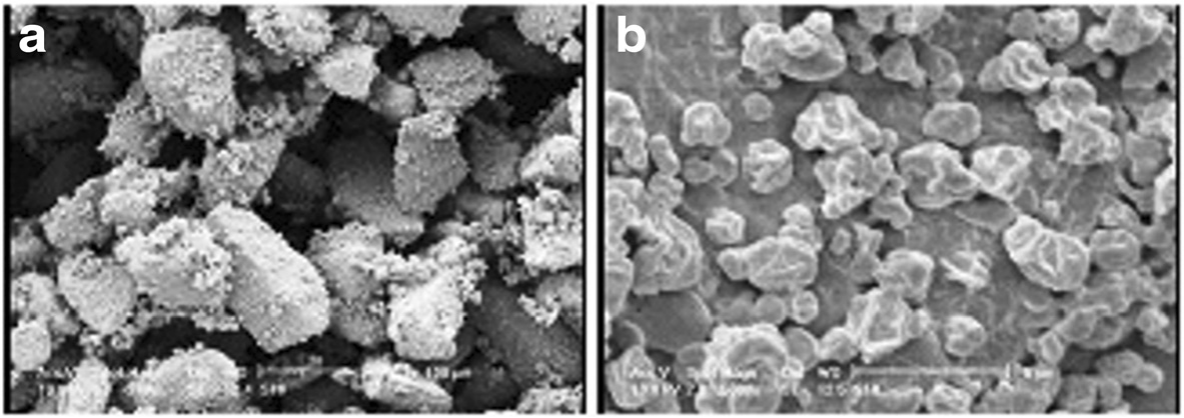
**Scanning electron micrographs of SLmPs blended with lactose. a)** magnification ×240, **b)** more magnification (×5000) representing SLmPs deposited on the surface of lactose carriers.

Scanning electron microphotographs of the SLmPs are shown in Figure 1. As shown in Figure 1a-c, changing the solvent in the feed solution did not seriously change the spherical shape of cholesterol-based SLmPs which is typically obtained via spray drying technique [32]. Processing of the drug and DPPC in ethanol produced particles similar to that of cholesterol-based samples (Figure 1d). However, as it is indicated in Figure 1e, applying a mixed solution of water-ethanol (30:70 v/v) in formulations consisted of DPPC resulted in production of wrinkled particles which used to be mostly spherical when pure ethanol was applied as the solvent. It is supposed that the solubility saturation of the formulation components upon former evaporation of the more volatile solvent (ethanol) leads to formation of a primary solid shell which then collapses as the core’s water content evaporates [33]. In this case, the surface-active DPPC could have contributed to the formation of this primary solid shell during particle formation stage. Incorporation of L-leucine within this formulation led the spherical shape back to the particles, as it is clearly shown in Figure 1f. It seems that the more tendency of L-leucine to water than ethanol and its subsequent localization in the core of the primary particles inhibited the shell to completely collapse after water evaporation. Figure 2 shows the attachment of SLmPs obtained from water-ethanol (30:70 v/v) solution of DPPC and SS to the large lactose surface. In fact, physical blending of the formulations with lactose monohydrate as the coarse carrier promoted the adhesion of SLmPs onto its surface. This process was expected to aid the deaggregation and dispersion of particles within the respiratory flow [34].

The true density values of the spray dried samples obtained by helium pycnometry are shown in Table 3. SS powders, which were spray dried from both kinds of the solvent systems, were used as controls. The results suggested that utilizing the lipid components along with the drug could result in reduction of the true density of the spray-dried powders. Actually, particle’s aerodynamic diameter (d_a_) is a function of particle’s geometric diameter (d), density (ρ) and morphology (χ, shape factor) according to the following equation:

**Table 3:** True density values obtained by the helium pycnometer

Drug conc. (%)*	**Excipients**	**Solvent system**	**Inlet temp. (°C)**	**Density (g/cm**^**3**^**)**
37.5	Cholesterol	Ethanol	80	1.11 ± 0.09
37.5	Cholesterol	Water/Ethanol	100	1.15 ± 0.10
37.5	DPPC	Ethanol	80	1.15 ± 0.08
37.5	DPPC	Water/Ethanol	100	1.18 ± 0.07
100	-	Ethanol	80	1.33 ± 0.11
100	-	Water/Ethanol	100	1.41 ± o.12

*Percentage of the total solid content (w/w).

$$d_{a}=d\surd \left(\rho /\chi \right)$$

In other words, particles with low density have smaller aerodynamic diameter than their geometric diameter. Thus, it can be of great value to reduce the density and affect the aerodynamic diameter of the particles by changing a DPI formulation composition. In this regard, Scalia et al. had previously reported the true density values of lower than 1 g cm^-3^ for the lipid microparticles obtained by melt emulsification technique [35].

### Aerosol performance of the SLmPs

Table 4 shows the ED (%), FPD (μg) and FPF (%) values of the spray dried SLmPs (formulations number 1 to 7) along with the same powders mixed with lactose carrier in the ratio of 1:9 w/w (formulations number 8 to 12). The aerodynamic characteristics were measured using a TSI at the flow rate of 60 L/min after aerosolization by Cyclohaler^®^. It should be noted that SS recoveries from the inhaler and the different parts of the TSI ranged between 90.1-95.2% of the total loaded drug.

**Table 4:** Fine particle dose (FPD), emitted dose (ED) and fine particle fraction (FPF) of salbutamol sulfate after aerosolization from different formulations (mean ± SD)

**Formulation number**	**Lipid excipients**	Solvent system**	**Inlet temp.(°C)**	Blending excipient**	**FPF(%)**	**FPD(**μ**g)**	**ED(%)**
1	Cholesterol	E	80	-	16.7 ± 0.8	165 ± 4.4	79.2 ± 2.1
2	Cholesterol	E	80	-	16.5 ± 1.2	305 ± 5.7	74.1 ± 2.5
3	Cholesterol	E	80	-	21.1 ± 0.9	575 ± 7.3	74.8 ± 1.8
4	DPPC	E	80	-	4.1 ± 0.3	138 ± 3.2	89.3 ± 1.6
5	Cholesterol	W/E	100	-	12.1 ± 0.7	310 ± 4.8	69.1 ± 2.1
6	DPPC	W/E	100	-	22.5 ± 1.3	686 ± 7.5	81.1 ± 2.3
7	DPPC + leucine	W/E	100	-	23.7 ± 1.1	712 ± 6.9	80.2 ± 1.9
8	Cholesterol	E	80	Lac.	24.1 ± 1.4	75 ± 3.1	82.6 ± 2.5
9	Cholesterol	W/E	100	Lac.	20.3 ± 0.8	61 ± 3.5	80.1 ± 2.2
10	DPPC	E	80	Lac.	16.6 ± 0.9	50 ± 2.8	80.1 ± 1.6
11	DPPC	W/E	100	Lac.	33.7 ± 1.5	108 ± 3.7	85.3 ± 2.7
12	DPPC + leucine	W/E	100	Lac.	42.7 ± 1.7	141 ± 4.1	87.9 ± 2.3
C_1_^*^	-	E	80	Lac.	17.6 ± 1.0	146 ± 2.8	83.4 ± 1.9
C_2_^*^	-	W/E	100	Lac.	14.4 ± 0.8	116 ± 2.2	81.1 ± 2.1

*C_1_ and C_2_ are control formulations of 5% (w/v) salbutamol sulfate in spray drying solution.

**E stands for Ethanol, W for Water, and Lac for lactose.

It seems that the type of solvent system and lipid excipients had a direct effect on aerosolization properties of the powders. Among the formulations prepared by cholesterol and ethanol, increasing the drug content from 12.5% to 25% did not make a significant change on FPF values (P > 0.05), but the initial drug content of 37.5% (Formulation No. 3) appeared to have higher FPF (%) than the others (P < 0.05). However, changing the type of cholesterol solvent to 30:70 v/v water-ethanol (Formulation No. 5) resulted in FPF reduction which seems to be due to particle size enlargement of the resultant SLmPs [36,37]. The difference between FPF values associated with the type of solvent was more noticeable when DPPC was used as the lipid excipient. The consequence of changing the solvent from pure ethanol to 30:70 v/v water-ethanol was a noticeable increase in FPF values from 4.1% to 22.5% for DPPC-based formulations (P < 0.05). The latter results are not in accordance with the particle size determinations obtained by laser diffraction, since the formulation prepared by the aid of ethanol solution of DPPC had smaller size than that of water-ethanol solution of it. In this case, the particle aggregation of very small particles (D50% =1.42 μm) made up of DPPC as the lipid excipient and ethanol as the solvent, seemed to be the main cause of owning the lowest FPF value. In addition, wrinkled particles usually improve the respirable fraction of a DPI formulation by decreasing the interparticulate cohesion forces as well as enhancing the powder dispersibility [38].

The incorporation of L-leucine to the formulation number 6 which was prepared from 30:70 v/v water-ethanol solution of DPPC and SS resulted in insignificant FPF improvement (P > 0.05). As mentioned earlier, both kinds of formulations (F6 and F7) had almost similar particle average diameters, but different shapes. Although L-leucine plays a role of anti-adherent amino acid that can improve the deagglomeration of SLmPs [29], it seems that the corrugated particles made from spray-dried SS and DPPC could compensate the absence of L-leucine and act as favorably as the spherical particles of F7 in the in vitro pulmonary deposition test.

Moreover, simple blending of micron-sized SLmPs with coarse lactose monohydrate terminated in noticeable FPF elevation, compared to the FPF values of uncombined SLmPs. It seems that the absorption of the SLmPs to the surface of lactose, and the subsequent improvement in the dispersibility and deaggregation of them within the airflow resulted in elevated drug deposition in stage 2 of the TSI [24,34]. Finally, we found that co spray-dried DPPC/L-leucine, which had then been blended with coarse lactose (in the ratio of 1:9 w/w), was the most appropriate formulation for SS in term of aerosol performance.

### In vitro drug release study

**Figure 3:**
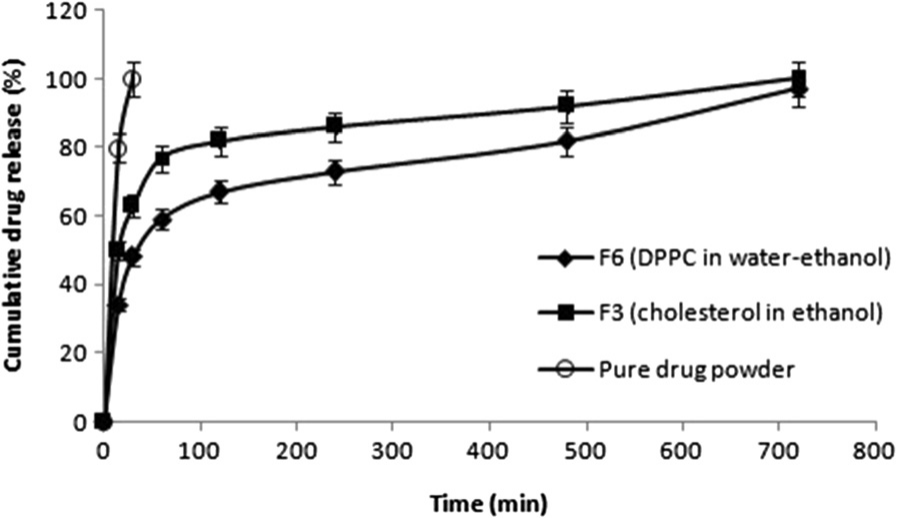
***In vitro *** **release profile of salbutamol sulfate from different formulations.**

The release profiles of SS from SLmPs are reported in Figure 3. It should be noted that release of pure micronized SS was rapid as nearly all the amount of the drug was released in less than 30 min, which is in accordance with other studies [35]. In this study, generating inhalable microspheres from SS, cholesterol and ethanol provided a SR profile of the drug. In this regard, several other studies had shown the usefulness of SLmPs in developing SR formulations [7,17,18]. As shown in Figure 3, the release profile of SS from SLmPs produced from cholesterol and ethanol exhibited a burst release of approximately 50%, followed by a sustained SS release pattern over 12 hours, whilst in cholesterol-based SLmPs obtained from water-ethanol solution of SS, no SR profile was observed. This observation can be explained by the fact that the drug has a hydrophilic and ionized nature and does not dissolve in ethanol, so upon application of water and ethanol as the mixed solvent system, the drug mainly partitions into the water phase during the particle formation stage in spray drying chamber, and thus accumulates on the surface of the particle as the water evaporates. However, when the ethanol suspension of the drug is used, it is more likely for SS to be entrapped within the core of SLmPs as it does not dissolve in ethanol and thus does not migrate to lipid surface of the generating microparticles. In contrast, DPPC-based microparticles from ethanol suspension of SS did not show any SR profile, while changing the feed solvent from ethanol to water-ethanol (30:70 v/v) improved the drug entrapment within these DPPC-based SLmPs and exhibited a SR profile over 12 hours with a burst release of nearly 35%. In fact, besides the effect of the solvent, the affinity between the drug and lipid material is another efficient factor, which determines the retention capacity of SLmPs [17]. Herein, DPPC tends to place at the surface of the particles while the drug mostly remains within the aqueous core of the primary particles in the drying chamber before all the water content is subjected to evaporation. Thus, it is possible for DPPC to serve as a SS-retarding carrier in the mentioned inhalable formulation.

It worth mentioning that, this kind of SR pattern should be justified according to the dissolution rate of the pure drug powder as well as its specific pulmonary delivery rout. In this regard, it can be an acceptable SR pattern for SS DPI formulation since the lung retention time of microparticles is dependent on the generation number of the airway where the inhaled particles are deposited, and our SLmPs showed high FPF indicating that they have the potential to sufficiently penetrate deep into the lungs and avoid mucociliary clearance in the conducting airways. So the prolonged duration of the effect of SS can be expected by the aid of these SLmPs.

## Conclusions

The type of lipid, presence of L-leucine in the feed solution, and the solvent system from which the SS-containing SLmPs were spray dried were the factors, which greatly affected the particle morphologies and aerosolization properties. We also observed substantial effects that physical mixing of spray-dried microparticles with coarse carrier can have on the aerosol performance. Among different DPI formulations, powders spray dried from water-ethanol solution of the drug, DPPC and L-leucine which were also physically blended with coarse lactose exhibited the best aerosolization properties. Despite having noticeable burst release during the first hour of the study, some SS-containing SLmPs showed significant release retardation compared the pure drug. The present study suggests that DPPC and L-leucine can be interesting additives for further developments of SS inhalable powder formulations.

## Competing interests

The authors declare that they have no competing interests.

## Authors’ contributions

ZD: Carried out the preparation and characterization of the DPI formulations and drafted the manuscript. KM: Supervisor andparticipated in drafting the manuscript. ARN: Supervisor. HRF: participated in analysis of the drug. MAB: participated in characterization of the powders. All authors read and approved the final manuscript.

## References

[B1] Courrier H Butz N Vandamme TF Pulmonary drug delivery systems: recent developments and prospects Crit Rev Ther Drug Carrier Syst 2002 19 no. 4 no. 5 10.1615/critrevtherdrugcarriersyst.v19.i45.40 12661699

[B2] Groneberg D Witt C Wagner U Chung K Fischer A Fundamentals of pulmonary drug delivery Resp Med 2003 97 382 387 10.1053/rmed.2002.1457 12693798

[B3] Labiris N Dolovich M Pulmonary drug delivery. Part I: physiological factors affecting therapeutic effectiveness of aerosolized medications Brit J Clin Pharmacol 2003 56 588 599 10.1046/j.1365-2125.2003.01892.x 14616418 PMC1884307

[B4] Zeng XM Martin GP Marriott C The controlled delivery of drugs to the lung Int J Pharm 1995 124 149 164 10.1016/0378-5173(95)00104-Q

[B5] Hardy JG Chadwick TS Sustained release drug delivery to the lungs Clin Pharmacokin 2000 39 1 4 10.2165/00003088-200039010-00001 10926347

[B6] Cook RO Pannu RK Kellaway IW Novel sustained release microspheres for pulmonary drug delivery J Control Rel 2005 104 79 90 10.1016/j.jconrel.2005.01.003 15866336

[B7] Schreier H Gonzalez-Rothi RJ Stecenko AA Pulmonary delivery of liposomes J Control Rel 1993 24 209 223 10.1016/0168-3659(93)90180-D

[B8] Lu D Hickey AJ Liposomal dry powders as aerosols for pulmonary delivery of proteins AAPS PharmSciTech 2005 6 E641 E648 10.1208/pt060480 16408866 PMC2750613

[B9] Abra R Mihalko PJ Schreier H The effect of lipid composition upon the encapsulation and in vitro leakage of metaproterenol sulfate from 0.2 μm diameter, extruded, multilamellar liposomes J Control Rel 1990 14 71 78 10.1016/0168-3659(90)90062-X

[B10] Parthasarathy R Gilbert B Mehta K Aerosol delivery of liposomal all-trans-retinoic acid to the lungs Cancer Chemother Pharmacol 1999 43 277 283 10.1007/s002800050895 10071977

[B11] Pilcer G Amighi K Formulation strategy and use of excipients in pulmonary drug delivery Int J Pharm 2010 392 1 19 10.1016/j.ijpharm.2010.03.017 20223286

[B12] Taylor KM Fan SJ Liposomes for drug delivery to the respiratory tract Drug Dev Ind Pharm 1993 19 123 142 10.3109/03639049309038764

[B13] Kellaway IW Farr SJ Liposomes as drug delivery systems to the lung Adv Drug Deliv Rev 1990 5 149 161 10.1016/0169-409X(90)90012-H

[B14] Niven RW Schreier H Nebulization of liposomes. I. Effects of lipid composition Pharm Res 1990 7 1127 1133 10.1023/A:1015924124180 2293210

[B15] Desai TR Hancock RE Finlay WH Delivery of liposomes in dry powder form: aerodynamic dispersion properties Eur J Pharm Sci 2003 20 459 467 10.1016/j.ejps.2003.09.008 14659490

[B16] Jaspart S Bertholet P Piel G Dogné J-M Delattre L Evrard B Solid lipid microparticles as a sustained release system for pulmonary drug delivery Eur J Pharm Biopharm 2007 65 47 56 10.1016/j.ejpb.2006.07.006 16962749

[B17] Sebti T Amighi K Preparation and in vitro evaluation of lipidic carriers and fillers for inhalation Eur J of Pharm Biopharm 2006 63 51 58 10.1016/j.ejpb.2005.11.003 16380243

[B18] Müller RH Mäder K Gohla S Solid lipid nanoparticles (SLN) for controlled drug delivery–a review of the state of the art Eur J Pharm Biopharm 2000 50 161 177 10.1016/S0939-6411(00)00087-4 10840199

[B19] Trotta M Cavalli R Carlotti M Battaglia L Debernardi F Solid lipid micro-particles carrying insulin formed by solvent-in-water emulsion–diffusion technique Int J Pharm 2005 288 281 288 10.1016/j.ijpharm.2004.10.014 15620868

[B20] Pilcer G Sebti T Amighi K Formulation and characterization of lipid-coated tobramycin particles for dry powder inhalation Pharm Res 2006 23 931 940 10.1007/s11095-006-9789-4 16715383

[B21] Masters K Spray drying handbook 1991 5 New York: Longman

[B22] Steckel H Brandes HG A novel spray-drying technique to produce low density particles for pulmonary delivery Int J Pharm 2004 278 187 195 10.1016/j.ijpharm.2004.03.010 15158961

[B23] Chow AH Tong HH Chattopadhyay P Shekunov BY Particle engineering for pulmonary drug delivery Pharm Res 2007 24 411 437 10.1007/s11095-006-9174-3 17245651

[B24] Son Y-J McConville JT Advancements in dry powder delivery to the lung Drug Dev Ind Pharm 2008 34 948 959 10.1080/03639040802235902 18800256

[B25] Sanna V Kirschvink N Gustin P Gavini E Roland I Delattre L Evrard B Preparation and in vivo toxicity study of solid lipid microparticles as carrier for pulmonary administration AAPS PharmSciTech 2004 5 17 23 10.1208/pt050227 PMC2750462 15760085

[B26] Nassimi M Schleh C Lauenstein H Hussein R Hoymann H Koch W Pohlmann G Krug N Sewald K Rittinghausen S A toxicological evaluation of inhaled solid lipid nanoparticles used as a potential drug delivery system for the lung Eur J Pharm Biopharm 2010 75 107 116 10.1016/j.ejpb.2010.02.014 20206256

[B27] Smolensky M D’alonzo G Kunkel G Barnes P Day-night patterns in bronchial patency and dyspnea: basis for once-daily and unequally divided twice-daily theophylline dosing schedules Chronobiol Int 1987 4 303 317 10.3109/07420528709083521 3315262

[B28] Chew NY Shekunov BY Tong HH Chow AH Savage C Wu J Chan HK Effect of amino acids on the dispersion of disodium cromoglycate powders J Pharm Sci 2005 94 2289 2300 10.1002/jps.20426 16136546

[B29] Salama RO Traini D Chan H-K Young PM Preparation and characterisation of controlled release co-spray dried drug–polymer microparticles for inhalation 2: Evaluation of in vitro release profiling methodologies for controlled release respiratory aerosols Eur J Pharm Biopharm 2008 70 145 152 10.1016/j.ejpb.2008.04.009 18534832

[B30] Al-Asheh S Jumah R Banat F Hammad S The use of experimental factorial design for analysing the effect of spray dryer operating variables on the production of tomato powder Food Bioprod Process 2003 81 81 88 10.1205/096030803322088215

[B31] Ståhl K Claesson M Lilliehorn P Lindén H Bäckström K The effect of process variables on the degradation and physical properties of spray dried insulin intended for inhalation Int J Pharm 2002 233 227 237 10.1016/S0378-5173(01)00945-0 11897427

[B32] Vehring R Pharmaceutical particle engineering via spray drying Pharm Res 2008 25 999 1022 10.1007/s11095-007-9475-1 18040761 PMC2292490

[B33] Lechuga‒Ballesteros D Charan C Stults CL Stevenson CL Miller DP Vehring R Tep V Kuo MC Trileucine improves aerosol performance and stability of spray‒dried powders for inhalation J Pharm Sci 2008 97 287 302 10.1002/jps.21078 17823950

[B34] Srichana T Brain A Marriott C Martin GP A study of drug-carrier interactions in dry powder inhaler formulations using the Andersen cascade impactor, X-ray microanalysis and time of flight aerosol beam spectrometry (TOFABS) Chem Pharm Bull 2000 48 167 174 10.1248/cpb.48.167 10705499

[B35] Scalia S Salama R Young P Traini D Preparation and in vitro evaluation of salbutamol-loaded lipid microparticles for sustained release pulmonary therapy J Microencap 2012 29 225 233 10.3109/02652048.2011.646326 22208706

[B36] Yu J Chien YW Pulmonary drug delivery: physiologic and mechanistic aspects Crit Rev Ther Drug Carrier Syst 1997 14 395 453 9450176

[B37] Bosquillon C Lombry C Preat V Vanbever R Comparison of particle sizing techniques in the case of inhalation dry powders J Pharm Sci 2001 90 2032 2041 10.1002/jps.1154 11745762

[B38] Zeng XM Martin GP Marriott C Particulate Interactions in Dry Powder Formulation for Inhalation 2000 London: Taylor & Francis

